# An intricate case of multidrug resistant *Plasmodium falciparum* isolate imported from Cambodia

**DOI:** 10.1186/s12936-017-1795-y

**Published:** 2017-04-14

**Authors:** Raffaele Dell’Acqua, Claudia Fabrizio, Francesco Di Gennaro, Sergio Lo Caputo, Annalisa Saracino, Michela Menegon, Mariangela L’Episcopia, Carlo Severini, Laura Monno, Francesco Castelli, Gioacchino Angarano

**Affiliations:** 1 Clinic of Infectious Diseases, Università degli Studi di Bari Aldo Moro, Piazza G. Cesare, 11, 70124 Bari, Italy; 2 Department of Infectious Diseases, Istituto Superiore di Sanità, Rome, Italy; 3 University Department of Infectious and Tropical Diseases, Azienda Ospedaliera Spedali Civili di Brescia, Brescia, Italy

**Keywords:** Malaria, Vivax, Malaria, Falciparum, Malaria, Drug resistance, Multiple, Antimalarials, Genes, MDR, Travel medicine, Cambodia

## Abstract

**Background:**

Imported cases of multidrug resistant *Plasmodium falciparum* and treatment failure with artemisinin-based regimens, although rare, have been described also in Western countries and their management is often challenging. This is also due to an inadequate knowledge and implementation of health prevention measures.

**Case report:**

A complex case of imported malaria caused by *Plasmodium vivax*/*P. falciparum* isolates in a patient who was not taking chemoprophylaxis while he was travelling in Cambodia is reported in this article. After failures of artemisinin-based and both oral and intravenous quinine-based regimens, a multidrug resistant *P. falciparum* was detected. The patient was successfully treated with atovaquone–proguanil.

**Conclusions:**

This experience highlights the importance of a careful management that should be based not only on the most up-to-date guidelines, but also on the awareness of a rapidly evolving scenario.

## Background

Imported cases of multidrug resistant (MDR) *Plasmodium falciparum* and treatment failure with artemisinin-based regimens, although rare, have been described also in Western countries and their management is often challenging [1, 2]. Even if uncommon, the possibility of imported infections by drug-resistant *Plasmodium* spp. should be considered, especially in travellers returning from highly endemic regions [3], where the most recent first-line artemisinin-based regimens are losing their effectiveness, also due to selective drug pressure [4, 5]. Moreover, the increasing accessibility to remote geographic areas of the world by do-it-yourself travellers not always keeps the pace with an adequate knowledge and implementation of health prevention measures. The following case illustrates these issues.

## Case presentation

A 27-years old male Italian patient, carrying thalassemia trait, returned on November 22nd, 2015 from a 4-week long pleasure trip to Cambodia, without taking any malaria chemoprophylaxis. This trip also included a 5-days trekking throughout the Pursat region. The day before his return, he had a rapid onset of fever up to 39 °C preceded by chills, cough and diarrhoea. Due to the persistence of fever despite therapy with ciprofloxacin and paracetamol, he was admitted to the Clinic of Infectious Diseases, Policlinico Hospital, Bari, on November 25th. Upon admission he had dehydration, mild leucocytosis (white blood cells 11.36 × 10^9^/L), haemoglobin (Hb) 13.3 g/dL, platelets (PLTs) 62 × 10^9^/L, C-reactive protein (CRP) 64.8 mg/L. On suspicion of malaria, peripheral blood smears and molecular biology testing (multiplex Real-Time PCR, Fast-Track Diagnostic) were performed, proving to be positive for *P. falciparum* and *Plasmodium vivax*, with a parasitaemia below 2%. A 3-day dihydroartemisinin–piperaquine regimen was initiated, with rapid defervescence and good clinical progress. After the end of treatment, blood smears resulted negative and the patient was discharged with prescription of 30 mg/day of primaquine for 14 days for radical cure.

On December 1th he was readmitted due to the reappearance of fever in the previous 2 days. Primaquine treatment had not been initiated due to delayed supply of the drug which is not readily available in Italy. Laboratory data showed anaemia (Hb 7.3 g/dL, requiring blood transfusion even in the absence of symptoms), thrombocytopaenia (PLTs 84 × 10^9^/L) and CRP elevation (40 mg/L). Peripheral blood smears were again positive for *P. falciparum* trophozoites; *P. vivax* gametocytes were also detected, as an expected biologic evolution without pathologic significance. This finding is consistent with missed start of primaquine. Therefore, a second-line therapy with oral quinine and doxycycline was started. A prompt clinical improvement was observed after 24 h; a 7-day course of therapy was completed leading to negative blood smears. Primaquine administration was withheld until the resolution of *P. falciparum* recrudescence and it was started on December 23rd shortly before hospital discharge. Indeed the patient was dismissed on December 24th with indication to complete a full 14-days course with weekly monitoring of complete blood count.

However, on January 18th the patient presented with fever and diarrhoea. After a few days of symptomatic home treatment with temporary benefit, he was readmitted showing thrombocytopaenia (PLTs 110 × 10^9^/L), anaemia (Hb 9.2 g/dL) and CRP elevation (40.3 mg/L). *Plasmodium falciparum* trophozoites were detected on blood smears and therapy was initiated with intravenous quinine (loading dose of 20 mg/kg, followed by maintenance dose of 10 mg/kg q8h for 7 days) at first and, subsequently, with atovaquone/proguanil, thus obtaining the definitive clearance of the parasite and healing.

The resistance of the *Plasmodium falciparum* isolate infecting this patient to anti-malarial drugs was assessed by the evaluation of single nucleotide polymorphisms (SNPs) of six molecular gene markers (*PfK13*, *Pfcrt*, *Pfmdr1*, *Pfdhfr*, *Pfdhps* and *PfCytB*) linked to resistance to artemisinin derivatives, quinolines, antifolates–cycloguanil and atovaquone.

Total DNA was extracted (PureLink Genomic DNA Kits-Invitrogen) from 200 µL of three patient’s blood specimens collected on November 26th, 2015 (first hospital admission), December 15th, 2015 (second admission) and January 29th, 2016 (third admission). The polymorphism of the *P. falciparum* K13-propeller gene, from codon 427 to codon 690, was assessed using the primers: ArtinnerF (GCCTTGTTGAAAGAAGCAGAA) and ArtouterR (CGCCATTTTCTCCTCCTGTA) and with PCR conditions described by Taylor et al. [6]. Analysis of *Pfcrt* and *Pfmdr1* genes was performed as previously reported [7, 8]. Different methods were used for the analysis of fragment of *Pfdhfr* gene spanning codons 51–108, *Pfdhps* domain (719 bp) and point mutation in *PfCytB* gene, according to Palmieri et al. [9], Menegon et al. [7] and Korsinczky et al. [10], respectively. All PCR products were sent to Eurofins Genomics Company (Germany) for sequencing, and sequences were compiled and analysed by Accelrys DS Gene software.

Analysis of polymorphisms of *P. falciparum* isolates showed a pattern of multidrug resistance due to the presence of point mutations associated with quinoline (including amodiaquine), drug resistance in *Pfcrt* and *Pfmdr1*, and mutations correlated to sulfadoxine/pyrimethamine resistance in *Pfdhps* and *Pfdhfr* genes (Table 1). In particular, in the *Pfk13* gene was observed the presence of the mutation C580Y, which is an important determinant of artemisinin resistance in *P. falciparum* population circulating in Southeast Asia [11, 12]. The absence of mutations at codons 258 and 268 of *CytB* gene indicated a sensitivity of the *P*. *falciparum* isolate to atovaquone and is consistent with the good response of the patient to atovaquone/proguanil administration.

**Table 1:**
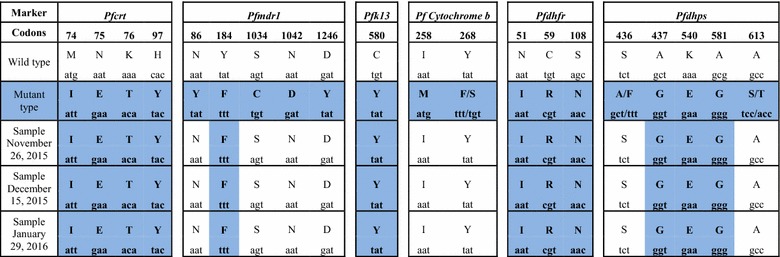
Analysis of polymorphisms of the *P. falciparum* isolates

*Pfcrt* and *Pfmdr1* genes point mutations are associated with quinoline (including amodiaquine) drug resistance; *Pfdhfr* and *Pfdhps* genes mutations are responsible for *P. falciparum* resistance to antifolate-cycloguanil; *Pfk13* gene mutation C580Y is considered an important determinant of artemisinin resistance

Mutant codons from the three different samples are in *blue*

## Discussion

This report shows the serious consequences, both in terms of clinical impact and health-related expenses, of a rare and extended resistance pattern of *P. falciparum* strain. Indeed, the management of this patient was complex; firstly, because he experienced three subsequent admissions, resulting in a prolonged hospitalization. Furthermore, once malaria recrudescence was confirmed, pharmacological treatment was cumbersome in terms of drug supply, costs, safety profile and consequent need of strict monitoring. An additional issue was represented by molecular analysis, which required the expertise of the Italian National Institute of Health (ISS) since these assays are not routinely available. Lastly, patient’s anxiety regarding the course of the disease should also be taken into account.

A double recrudescence of *P. falciparum* in the same patient is a rare event to observe. Moreover, the chance of a recrudescence after a second-line treatment of oral quinine plus doxycycline is very low [13]. Reasons for failure with this regimen may include a decrease in the sensitivity or an inadequate exposure to the drug caused by unusual pharmacokinetics in an individual, scarce adherence to the prescribed regimen or poor quality of anti-malarial drugs [14]. In this case, therefore, the concurrence of both pathogen- and host-related factors could have been responsible for the second recrudescence.

Based on the multidrug resistance pattern of *P. falciparum*, a complicated clinical course could have been expected. However, with the exception of a single blood transfusion, the patient did not require any extraordinary therapeutic measure. Thalassaemia trait probably played a relevant role in attenuating the severity of the disease, whereas the clinical impact of dual infection was unclear, based on the heterogeneous results of several studies regarding the mutual interactions between the two Plasmodium species and the role of immunity [15].

As Cambodia is known to be the cradle of anti-malarial drug resistance, patients returning from this area should be considered at risk for failure of artemisinin-based regimens [16]. Therefore, according to our experience, even the most up-to-date guidelines should be handled with care in this rapidly evolving scenario and to this regard, the atovaquone/proguanil combination may be considered as a valuable therapeutic option in some special cases [17].

In a context of increasing international travel and trades in which exotic regions are easier to reach, the relevance of a proper prophylaxis should be highlighted in order to obtain individual protection. Moreover, in this framework, an increased awareness about the possibility to manage such difficult cases also in non-endemic settings should become a matter of utmost importance.

## Abbreviations

MDR: multidrug resistant
Hb: haemoglobin
PLTs: platelets
CRP: C-reactive protein
SNPs: single nucleotide polymorphisms
ISS: Istituto Superiore di Sanità
